# Perspectives for therapeutic HPV vaccine development

**DOI:** 10.1186/s12929-016-0293-9

**Published:** 2016-11-04

**Authors:** Andrew Yang, Emily Farmer, T. C. Wu, Chien-Fu Hung

**Affiliations:** 1 Department of Pathology, Johns Hopkins University, Baltimore, MD USA; 2 Department of Obstetrics and Gynecology, Johns Hopkins University, Baltimore, MD USA; 3 Department of Molecular Microbiology and Immunology, Johns Hopkins University, Baltimore, MD USA; 4 Department of Oncology, Johns Hopkins University, Baltimore, MD USA; 5 The Johns Hopkins University School of Medicine, CRB II Room 307, 1550 Orleans Street, Baltimore, MD 21231 USA

**Keywords:** Human papillomavirus, HPV, Therapeutic vaccine, Cervical cancer, HPV E6, HPV E7

## Abstract

**Background:**

Human papillomavirus (HPV) infections and associated diseases remain a serious burden worldwide. It is now clear that HPV serves as the etiological factor and biologic carcinogen for HPV-associated lesions and cancers. Although preventative HPV vaccines are available, these vaccines do not induce strong therapeutic effects against established HPV infections and lesions. These concerns create a critical need for the development of therapeutic strategies, such as vaccines, to treat these existing infections and diseases.

**Main Body:**

Unlike preventative vaccines, therapeutic vaccines aim to generate cell-mediated immunity. HPV oncoproteins E6 and E7 are responsible for the malignant progression of HPV-associated diseases and are consistently expressed in HPV-associated diseases and cancer lesions; therefore, they serve as ideal targets for the development of therapeutic HPV vaccines. In this review we revisit therapeutic HPV vaccines that utilize this knowledge to treat HPV-associated lesions and cancers, with a focus on the findings of recent therapeutic HPV vaccine clinical trials.

**Conclusion:**

Great progress has been made to develop and improve novel therapeutic HPV vaccines to treat existing HPV infections and diseases; however, there is still much work to be done. We believe that therapeutic HPV vaccines have the potential to become a widely available and successful therapy to treat HPV and HPV-associated diseases in the near future.

## Background

Cervical cancer is the fourth most common cancer effecting women worldwide [1]. Human papillomavirus (HPV) accounts for nearly all cases of cervical cancer and is responsible for causing several other cancers including: penile, vaginal, vulval, anal and oropharynx including base of the tongue and tonsils [1–4]. There are over 200 types of HPV [5], which are categorized into high risk, and low risk groups according to their oncogenic potential [6, 7]. Among high risk HPV types, type 16 and type 18 are the most common and carcinogenic. Combined, these two HPV types are responsible for about 70 % of cervical cancer cases [8].

Identifying HPV as the etiological factor for HPV-associated malignancies has created the opportunity to control those cancers through vaccination and other therapeutic strategies [9]. Vaccines have been traditionally used as a prophylactic measure against infectious diseases. Several successful prophylactic HPV vaccines have been developed targeting the major capsid protein L1 of the viral particle (for review see [10, 11]). Prophylactic vaccines have been successful at preventing healthy patients from acquiring HPV infections as well as previously infected patients from being re-infected; however, they are not able to treat or clear established HPV infections and HPV-associated lesions (for review see [10, 12]). One potential treatment method that has been explored to treat and clear existing HPV infections and associated diseases are therapeutic HPV vaccines. Unlike prophylactic HPV vaccines, which are used to generate neutralizing antibodies against viral particles, therapeutic HPV vaccines are used to stimulate cell-mediated immune responses to specifically target and kill infected cells.

Most sexually active women will be infected by HPV at some point in their life. For many women these infections remain asymptomatic and are cleared by the immune system. However, some women can develop persistent HPV infections, which may further develop into low or high-grade cervical intraepithelial neoplasia (CIN) and cervical carcinoma, or regress at any stage [13, 14]. In many HPV-associated lesions that progress into cancers, the HPV viral DNA genome are found to be integrated into the host’s genome. This process often leads to the deletion of many early (E1, E2, E4, and E5) and late (L1 and L2) genes. The deletion of L1 and L2 during the integration process is what renders prophylactic vaccines useless against HPV-associated cancers. In addition, E2 is a negative regulator for the HPV oncogenes E6 and E7. The deletion of E2 during integration leads to elevated expression of E6 and E7 and is thought to contribute to the carcinogenesis of HPV-associated lesions (for review see [9, 15]). Oncoproteins E6 and E7 are required for the initiation and upkeep of HPV-associated malignancies and are resultantly expressed and present in transformed cells [16]. Furthermore, therapeutic HPV vaccines targeting E6 and E7 can circumvent the problem of immune tolerance against self-antigens because these virus encoded oncogenic proteins are foreign proteins to human bodies. For these reasons HPV oncoproteins E6 and E7 serve as an ideal target for therapeutic HPV vaccines [12].

Although prophylactic HPV vaccines have been a huge success and leap forward in the prevention of HPV infections and HPV-associated diseases, there is still a great HPV-associated disease burden worldwide. As such, there is an urgent need to develop treatments for the control and eradication of existing HPV infections and associated diseases. Our review will cover various therapeutic HPV vaccines in development for the treatment of HPV infections and HPV-associated diseases, including HPV-associated cancers. In addition, we will focus on the findings of latest clinical trials on therapeutic HPV vaccines.

### Types of therapeutic HPV vaccines

**Fig. 1:**
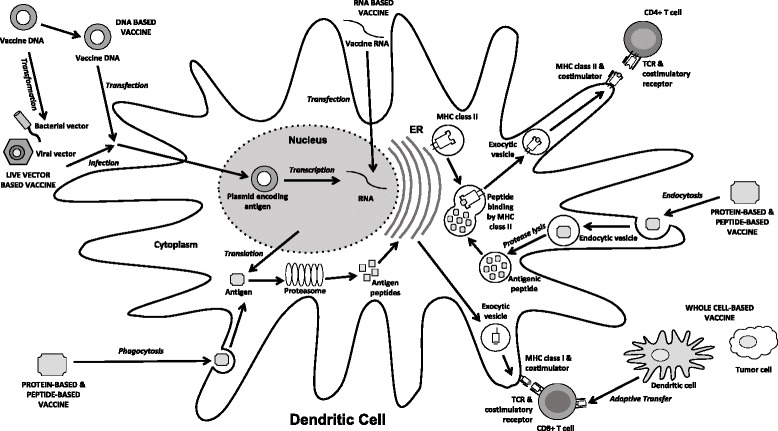
Immune activation by therapeutic HPV vaccination. Administration of varying therapeutic HPV vaccine types results in the delivery of different forms of antigen into the body. DNA plasmids encoding HPV oncoproteins E6 and E7 can be transfected into dendritic cells through DNA vaccines or infection of transformed live vector-based vaccines. These antigens are then transcribed into RNA; however, RNA can also be introduced into the cell through RNA vaccines. Transcribed RNA is further translated into antigen proteins or long peptides. Antigen proteins or long peptides can also be taken up by the dendritic cell through phagocytosis after administration of a protein-based or peptide-based vaccine. These proteins or peptides are processed into short peptides by proteasomes and loaded onto an MHC class I molecule in the endoplasmic reticulum (ER) to be presented to T cell receptors on CD8+ T cells. In addition, dendritic cells or tumor cells can be prepared ex vivo to express target antigens on MHC class I molecules with necessary co-stimulatory molecules and be administered back into the body as whole cell-based vaccines through adoptive transfer in order to prime T cells. On the other hand, the protein or peptide antigens taken up by the dendritic cell can be degraded into smaller fragments by proteases in the endosome. The endosome containing the small antigenic peptides is then fused with the exosome containing MHC class II molecule, during which the antigenic peptide is loaded onto the MHC class II molecule. The MHC class II – antigenic peptide complex is then transported to the cell surface to be presented to T cell receptors on CD4+ T cells

Several types of therapeutic vaccines have been developed and tested in preclinical and clinical trials, including live vector, protein or peptide, nucleic acid, and cell-based vaccines (for review see [16]). Importantly, clinical trials are necessary to evaluate whether a therapeutic HPV vaccine is able to control HPV infections and HPV-associated diseases in humans. The majority of these vaccines target HPV oncoproteins E6 and E7 with the aim to deliver E6 and E7 antigens in various forms to antigen presenting cells (APCs) in order to activate HPV antigen-specific CD8+ cytotoxic T cells or CD4+ helper T cells, respectively (Fig. 1). Importantly, E6 and E7 antigens need to be processed and digested by proteasomes into smaller peptides before they can be presented on the MHC class I molecule of the APCs for the activation of CD8+ T cells. However, not all peptide fragments from the antigenic proteins are loaded on MHC molecules and recognized by antigen-specific T cells [17]. Only a selected few of these short peptides contain the sequence of antigenic fragments (epitopes) that can bind to the MHC molecule with high affinity and subsequently interact with the T cell receptor (TCR) of antigen-specific T cells to elicit an immune response [18–20]. Most therapeutic vaccines have been designed to elicit an immune response against the E7 antigen because it is better characterized immunologically than the E6 antigen in preclinical models.

The following section discusses the characteristics of various therapeutic HPV vaccines being developed and tested. The section summarizes numerous recent clinical trials that have been implemented using various types of therapeutic HPV vaccines against HPV-associated lesions and malignancies. Table 1 summarizes the clinical trials on therapeutic HPV vaccines described in this section. In addition, Table 2 lists several ongoing clinical trials evaluating the efficacy of therapeutic HPV vaccines against HPV-associated diseases.

**Table 1:** Different forms of therapeutic HPV vaccines recently used in clinical trials

Vaccine	Antigen(s)	Construct	Organization	Trial design	Outcome	Side effects	Reference
Bacterial Vector Based							
Lm-LLo-E7 (ADXS11-001; ADXS-HPV)	HPV-16 E7	prfA-defective Listeria monocytogenes strain transformed with plasmid encoding HPV-16 E7 antigen fused to a fragment of nonhemolytic listeriolysin O (LLO)	Advaxis, Inc.	Phase I in patients with metastatic, refractory or recurrent, advanced squamous cell carcinoma of the cervix (15 patients)	Increase in E7-specific T cells detected in PBMCs of three patients. Reduction in tumor size observed in 4 patients.	Pyrexia, vomiting, chills, headache, anemia, nausea, tachycardia, muscle and skeletal pain.	[30]
GLBL101c	HPV16-E7	Recombinant *Lactobacillus casei* expressing modified version of HPV16-E7	GENOLAC BL Corp	Phase I/IIa in HPV16+ CIN3 patients (17 patients)	Significant increase in E7-CMI in cervical vaginal tract. 9 patients experienced disease regression to CIN2, and 5 further regressed to LSIL	No major side effects observed.	[32]
Viral Vector Based							
TA-HPV	HPV-16/18 E6/E7	Recombinant Vaccinia virus	European Organization for Research and Treatment of Cancer (EORTC)	Phase I/II in patients with advanced stage of cervical cancer (8 patients)	Vaccination induced HPV-specific cytotoxic T lymphocyte immune response in 28 % of participants (3 out of 8). 2 patients showed tumor free condition at 15 and 21 months after vaccination.	Single dose generated mild and tolerable toxicity	[46]
				Phase I in patients with clinical International Federation of Gynecology and Obstetrics (FIGO) stage Ib or IIa cervical cancer who will undergo radical hysterectomy (29 patients)	After a single vaccination HPV-specific CTLs were found in 4 patients. 8 patients (28 %) developed HPV-specific serological responses.	Mild to moderate local toxicity	[54]
				Phase II in patients ages 42–54 with high-grade HPV-positive vulval or vaginal intraepithelial neoplasia of up to 15 years duration (12 patients)	5 of 12 (42 %) patients showed at least 50 % reduction in total lesion diameter over 24 weeks with 1 patient showing complete regression of lesion. Overall, 83 % of women showed some average decrease in lesion size of 40 %. All patients showed an increased IgG titer and T-cell response to the vaccinia virus.	A local reaction at the site of vaccination between day 7–10 was common and 2 patients had temporarily limited arm movement.	[55]
TG4001	HPV-16 E6/E7	Recombinant modified vaccinia Ankara-expressing HPV-16 E6, E7, and IL-2	Transgene/roche	Phase I in HPV16+ CIN2/3 patients (21 patients)	Ten of 21 (48 %) showed disease regression, HPV DNA clearance in eight patients and mRNA clearance in seven patients	Inflammation, pruritus, edema, lymphadenopathy, fever, headache, asthenia, bone pain, vaginal discharge	[47]
MVA E2	HPV-16 E2	Recombinant Modified Vaccinia Ankara encoding E2 from BPV	Instituto Mexicano del Seguro Social	Phase III in patients with HPV-induced AGIN (1176 female patients and 180 male patients)	90 % lesion clearance in female treated patient and 100 % lesion clearance in male treated patients. Antibody and T cell responses observed in all tested patients.	Headache, flu-like symptom, fever, chills, abdominal pain, joint pain.	[53]
Peptide/Protein Based							
HPV16-SLP	HPV-16 E6/E7	Combination of nine HPV-16 E6 and four HPV-16 E7 synthetic peptides with incomplete Freund’s adjuvant	ISA Pharmaceuticals	Phase II in patients with HPV16+ VIN3 (20 patients)	15 patients had objective clinical response at 12 months. 9 complete responses and 6 partial response. 85 % with circulating HPV-16 specific T cells. 83 % had CMI against HPV-16.	Local swelling, redness, increased skin temperature, pain at vaccination site, fever, flu like symptoms, chills, and tiredness.	[60]
				Phase II study in patients with HPV16+ HSIL (9 patients)	All vaccinated patients showed strong HPV-specific T cell response after vaccination. Change in patterns of immune infiltrate.	Itching, redness, swelling, and pain at injection site, headache, diarrhea, fatigue/dizziness, nausea, chills, myalgia, rash, fever, urticarial, edema.	[59]
				Phase II in patients HPV16+ advanced or recurrent gynecological carcinoma (20 patients)	9 patients with HPV-16 specific immune response. Duration of survival correlates with magnitude of T cell response.	Injection site reaction, fever, chills, fatigue, nausea, flue-like symptom	[61]
				Phase II in patients with low-grade abnormalities of the cervix (50 patients)	97 % of vaccinated patients generated HPV 16-specific CMI	Flu-like symptom, injection site reaction.	[63]
				Observational study in patients with advanced, recurrent, or metastatic cervical cancer scheduled to receive standard Carboplatin/Paclitaxel chemotherapy (18 patients).	11 of 12 vaccinated patients induced proliferative T cell responses	Chemotherapy related anemia, thrombocytopenia, leucopenia, neutropenia, and alopecia. Cancer related shortness of breath, pulmonary embolism, abdominal pain, gastroenteritis, erysipelas, hydronephrosis	[64]
GL-0810	HPV-16 antigen	HPV-16 immunomodulatory peptide with adjuvant Montanide and GM-CSF	Gliknik Inc.	Phase I in patients with recurrent/metastatic squamous cell carcinoma of the head and neck (5 patients)	80 % of patients who received four vaccinated developed T cell and antibody response. Progression free and overall survival is 8- to 196 days respectively	Erythema, itching, and pain at injection site	[66]
Pepcan + Candin	HPV-16 E6	HPV16 E6 peptides combined with Candida skin testing reagent candin.	University of Arkansas	Phase I study in patients with biopsy-confirmed HSIL (31 patients)	45 % patients experienced histological disease regression.	Mild to moderate injection site reaction.	[65]
GTL001 (ProCervix)	HPV-16 and HPV-18	Recombinant HPV16 and HPV18 E7 proteins fused to catalytically inactive *Bordetella pertussis* CyaA expressed in *E. coli*	Genticel	Phase I trial in patients positive for HPV-16 or HPV-18 infection but with normal cytology (47 patients)	Patients in cohort 4 (*n* = 9) who received 600ug GTL001 powder + imiquimod experienced the highest HPV16/18 clearance rate.	Injection site reactions including pain, swelling, induration, tenderness, and itching	[73]
TA-CIN	HPV-16 E6/E7/L2	HPV16 E6E7L2 fusion protein	Xenova Research Limited	Phase I in healthy patients (40 subjects)	TA-CIN specific IgG in 24 of 32 vaccinated patients. 25 of 32 vaccinated patients generated CMI.	Injection site reaction, tenderness. Headache and fatigue	[70]
				Phase II with VIN2/3 patients (19 patients)	63 % lesion response 1 year after vaccination. Significant increase. Significant CMI observed in lesion responders.	Local reaction associated with imiquimod.	[72]
TA-CIN + TA-HPV	HPV-16/18 E6/E7/L2	HPV16 E6E7L2 fusion protein and vaccinia virus with HPV16/18 E6/E7	Celtic Pharma	Phase I with HPV16+ VIN patient (10 patients)	Partial or complete clinical response in 2 patients. All but 1 patient showed HPV-16 specific IgG and/or T cell responses.	Pain at injection site.	[69]
				Phase II with HPV16+ high-grade AGIN Patients (29 patients)	17 patients showed TA-CIN induced T cell responses. 11 generated HPV-16/18 E6 and/or E7 specific T cells. 14 with IgG response to HPV-16 E7.	N/A	[71]
Nucleotide Based							
pNGVL4a-sig/E7(detox)/HSP70 + TA-HPV	HPV-16/18 E6/E7	Plasmid encoding mutated form of HPV16-E7 linked to sig and HSP70 and vaccinia virus with HPV16/18 E6/E7	Sidney Kimmel Comprehensive Cancer Center	Phase I with HPV16+ CIN3 Patients (12 patients)	58 % vaccinated patients have generated HPV-16 E7-specific CMI. Increase CD8+ T cell infiltration to lesions.	Tenderness, local site reaction, blister, erythema, pruritus	[79]
pNGVL4a-CRT/E7(detox)	HPV-16 E7	Plasmid encoding mutated form of HPV16-E7 linked to CRT	Sidney Kimmel Comprehensive Cancer Center	Phase I with HPV16+ CIN2/3 Patients (32 patients)	30 % vaccinated patients experienced histological regression to CIN1 or less. Increase in intraepithelial C8+ T cells infiltrate after vaccination.	Injection site reaction.	[81]
GX-188E	HPV-16/18 E6/E7	Plasmid encoding fusion protein of HPV 16/18 E6/E7 linked to Flt3L and tpa	Genexine, Inc	Phase I in patients with HPV 16/18+ CIN3 (9 patients)	All patients displayed enhanced HPV-specific CMI. 7 patients demonstrated complete lesion regression by the end of the trial.	Chills, injection site pain, swelling, hypoesthesia, headache, fatigue, rhinitis	[82]
VGX-3100	HPV-16/18 E6/E7	Mixture of two plasmids encoding optimized consensus of E6 and E7 antigen of HPV 16 and 18	Inovio Pharmaceuticals	Phase I with HPV16/18 + CIN2/3 Patients (18 patients)	HPV-specific CMI observed in 78 % patients and HPV-specific humoral response observed in all patients.	Injection site reaction, pain, fever, tenderness.	[85]
				Phase IIb with HPV16/18 + CIN2/3 Patients (167 patients)	49.5 % vaccinated patient demonstrated regression compared to 30.6 % in placebo group. Vaccinations enhance T cell and humoral response.	Injection site reaction, fatigues, headache, lyalgia, nausea, arthralgia, erythema	[86]
Whole Cell Based							
DC + KLH	HPV-16 and HPV-18 E7	Dendritic Cells pulsed with HPV-16 and HPV-18 E7 and keyhole limpet hemocyanin	National Institutes of Health	Phase I in patients with stage Ib or IIa cervical cancer (10 patients)	Increase in HPV-specific humoral and CD4+ T cell responses observed, but not CD8+ T cell responses.	Local site reaction, erythema, swelling, pruritus	[101]
DC	HPV antigens	DC pulsed with HPV+ tumor lysate	Department of Biotechnology (DBT, Govt. of India)	Phase I in in patients with HPV+ advanced, recurrent cervical cancer (14 patients)	No significant increase in lymphocyte proliferation observed. Lack of biopsy sample and small sample size prevent definite conclusions.	Local site reaction, fever, chills, abdominal discomfort, vomiting.	[102]

*CIN* Cervical intraepithelial neoplasia, *AGIN* Ano Genital Intraepithelial Neoplasia, *HSIL* High-grade squamous intraepithelial lesion, *VIN* vulvar intraepithelial neoplasia

**Table 2:** Ongoing therapeutic HPV vaccine clinical trials

Vaccine	Antigen(s)	Construct	Organization	Trial Design	Estimated Date of Trial Completion	Clinical Trials.gov Identifier
Persistent HPV Infection and Low-Grade Squamous Intraepithelial Lesion						
PDS0101	HPV-16 E6/E7	R-enantiomer of 1,2-dioleoyl-3-trimethylammonium-propane chloride + Peptides HPV-16 E6 and E7	PDS Biotechnology Corp.	Phase I in female patients with high risk HPV infection or CIN1 (18 estimated patients)	Information not provided	NCT02065973
ProCervix	HPV-16/18 E7	2 recombinant adenylate cyclase (CyaA) proteins: CyaA-HPV 16E7 & CyaA-HPV 18E7	Genticel	Phase II in female patients with HPV16/18+ infection or ASCUS/LSIL (220 estimated patients)	December 2016	NCT01957878
Cervical Intraepithelial Neoplasia (CIN)/High-Grade Squamous Intraepithelial Lesion						
GX-188E	HPV-16/18 E6/E7	Plasmid encoding fusion protein of HPV 16/18 E6/E7 linked to Flt3L and tpa	Genexine, Inc	Phase II in HPV 16/18+ CIN2, CIN2/3, and CIN3 patients in Eastern Europe (120 estimated patients)	December 2017	NCT02596243
				Phase II in HPV 16/18+ CIN3 patients in South Korea (72 estimated patients)	October 2016	NCT02139267
pNGVL4a-CRT/E7(detox)	HPV-16 E7	Plasmid encoding mutated form of HPV16-E7 linked to calreticulin	Sidney Kimmel Comprehensive Cancer Center	Phase I in patients with HPV16+ CIN2/3 (39 estimated patients)	March 2017	NCT00988559
pNGVL4a-sig/E7(detox)/HSP70 + TA-HPV	HPV-16/18 E6/E7	Plasmid encoding mutated form of HPV16-E7 linked to sig and HSP70 and vaccinia virus with HPV16/18 E6/E7		Phase I in patients with HPV16+ CIN3 in combination with topical imiquimod (48 estimated patients)	December 2016	NCT00788164
TVGV-1 + GPI-0100	HPV-16 E7	Fusion protein of HPV-16 E7 and ER targeting sequence	THEVAX Genetics Vaccine Co.	Phase IIa in patients with HPV induced cervical HSIL (51 estimated patients)	June 2017	NCT02576561
Pepcan + Candin	HPV-16 E6	HPV16 E6 peptides combined with Candida skin testing reagent candin.	University of Arkansas	Phase II in patients with cervical HSIL (125 estimated patients)	August 2020	NCT02481414
Anal Intraepithelial Neoplasia (AIN)						
ISA101 (SLP-HPV-01; HPV16-SLP)	HPV-16 E6/E7	Combination of nine HPV-16 E6 and four HPV-16 E7 synthetic peptides with incomplete Freund’s adjuvant	ISA Pharmaceuticals	Phase I/II in HIV+ male patients with HPV-16+ AIN2/3 (45 estimated patients)	February 2018	NCT01923116
ADXS11-001 (Lm-LLo-E7)	HPV-16-E7	prfA-defective Listeria monocytogenes strain transformed with plasmid encoding HPV-16 E7 antigen fused to a fragment of nonhemolytic listeriolysin O (LLO)	Advaxis, Inc.	Phase II trial in patients with persistent, recurrent, loco-regional, or metastatic anal cancer or HPV+ squamous cell carcinoma of the rectum that are either treatment naïve in the metastatic setting or have progressed or become intolerant to platinum based therapy (55 estimated patients)	March 2017	NCT02399813
HPV-Associated Incurable Solid Tumors						
ISA101 (SLP-HPV-01; HPV16-SLP)	HPV-16 E6/E7	Combination of nine HPV-16 E6 and four HPV-16 E7 synthetic peptides with incomplete Freund’s adjuvant	ISA Pharmaceuticals	Phase II in patients with HPV-16+ Incurable solid tumors (oropharyngeal squamous cell carcinoma, cervical, vulvar, vaginal, anal, and penile cancer) as combination therapy with Nivolumab (28 estimated patients)	December 2018	NCT02426892
DPX-E7	HPV-16 E7	HPV16-E711-19 nanomer	Dana-Farber Cancer Institute	Phase Ib/II trial in HLA-A*02 positive patients with incurable HPV 16-related oropharyngeal, cervical and anal cancer (44 estimated patients)	May 2023	NCT02865135
Head and Neck Cancer						
ADXS11-001 (Lm-LLo-E7)	HPV-16-E7	prfA-defective Listeria monocytogenes strain transformed with plasmid encoding HPV-16 E7 antigen fused to a fragment of nonhemolytic listeriolysin O (LLO)	Advaxis, Inc.	Phase II in patients with HPV+ Oropharyngeal Squamous Cell Carcinoma before robot-assisted resection (30 estimated patients)	March 2017	NCT02002182
				Phase I/II in patients with locally advanced or metastatic cervical or HPV+ Head and nack cancer with or without MED14736 chemoblie (66 estimated patients)	December 2019	NCT02291055
INO-3112 (VGX-3100 + INO-9012)	HPV-16/18 E6/E7	Mixture of three plasmids encoding optimized consensus of E6 and E7 antigen of HPV 16 and 18 and proprietary immune activator expressing IL-12	Inovio Pharmaceuticals	Phase I/IIA in patients with HPV associated head and neck squamous cell carcinoma (25 estimated patients)	December 2017	NCT02163057
Cervical Cancer						
ADXS11-001 (Lm-LLo-E7)	HPV-16-E7	prfA-defective Listeria monocytogenes strain transformed with plasmid encoding HPV-16 E7 antigen fused to a fragment of nonhemolytic listeriolysin O (LLO)	Advaxis, Inc.	Phase II in patients with persistent or recurrent squamous or non-squamous cell carcinoma of the cervix (67 estimated patients)	October 2018	NCT01266460
INO-3112 (VGX-3100 + INO-9012)	HPV-16/18 E6/E7	Mixture of three plasmids encoding optimized consensus of E6 and E7 antigen of HPV 16 and 18 and proprietary immune activator expressing IL-12	Inovio Pharmaceuticals	Phase I/IIA in female patients with new, recurrent, or persistent cervical cancer (30 estimated patients)	April 2019	NCT02172911
				Phase II in patients with locally advanced cervical cancer as combination therapy with chemoradiation (126 estimated patients)	May 2021	NCT02501278
ISA101 (SLP-HPV-01; HPV16-SLP)	HPV-16 E6/E7	Combination of nine HPV-16 E6 and four HPV-16 E7 synthetic peptides with incomplete Freund’s adjuvant	ISA Pharmaceuticals	Phase I/II in female patients with HPV-16+ advanced or recurrent cervical cancer (48 estimated patients)	December 2016	NCT02128126
TA-CIN + GPI-0100	HPV-16 E6/E7/L2	HPV16 E6E7L2 fusion protein + GPI-0100 adjuvant	Sidney Kimmel Comprehensive Cancer Center	Phase I in patients with HPV16 associated cervical cancer (30 estimated patients)	May 2020	NCT02405221

*AGIN* Ano Genital Intraepithelial Neoplasia, *AIN* Anal intraepithelial Neoplasia, *ASCUC* atypical squamous cells of undetermined significance, *CIN* Cervical intraepithelial neoplasia, *ER* Endoplasmic reticulum, *HIV* Human immunodeficiency virus, *HPV* Human papillomavirus, *HSIL* High-grade squamous intraepithelial lesion, *LSIL* Low-grade squamous intraepithelial lesion

#### Live vector-based vaccines

Live vector-based vaccines are often categorized as either bacterial or viral vectors depending on their vector platform. These vectors replicate within the body and facilitate the spread of the antigen [12, 16]. Live vector-based therapeutic HPV vaccines are highly immunogenic and can induce strong cellular and humoral immune responses (for review see [16]). They can also deliver E6 and E7 antigens to APCs to stimulate antigen presentation through MHC class I and II. Unfortunately, live vector-based vaccines pose a potential safety risk, particularly in immunocompromised individuals [12]. Additionally, the immune response efficacy after repeated immunization using the same vector is limited [12, 21, 22].

##### Bacterial vectors

Several bacterial vectors have been selected for the development of therapeutic HPV vaccines including *Listeria monocytogenes*, *Lactobacillus lactis*, *Lactobacillus plantarum*, and *Lactobacillus casei* [23–26]. Listeria has been recognized as a promising vector because of its ability to infect macrophages and secrete listeriolysin O (LLO), a pore-forming toxin, to evade phagosomal lysis [27]. Because it is able to evade phagosomal lysis, Listeria is able to replicate in the cytoplasm of the host cell. This ability further allows the bacteria to be present in both cytoplasm and endosomal compartments allowing the antigen peptides in the bacteria to be presented on both MHC class I to cytotoxic T cells and MHC class II to T helper cells [12, 26, 28, 29].

The first clinical use of a listeria-based therapeutic HPV vaccine was reported in 2009 [30]. The vaccine Lm-LLO-E7 (also known as ADXs11-001 or ADXS-HPV) contains prfA-defective Lm strain, transformed with HPV16 E7 antigen and a fragment of nonhemolytic LLO [31]. The phase I trial tested the safety of Lm-LLO-E7 in 15 patients with metastatic, refractory, or recurrent advanced squamous cell carcinoma of the cervix. Patients received the vaccine intravenously, followed by IV supplementation of 500 mg of ampicillin 5 days after vaccination, followed by a 10-day oral course of ampicillin (500 mg). The vaccine was well tolerated by patients; common adverse effects included pyrexia, vomiting, chills, headache and anemia, nausea and tachycardia, and musculoskeletal pain. Furthermore, six patients experienced vaccine-related grade 3 adverse events. Peripheral blood mononuclear cells (PBMCs) were collected from patients and tested, showing an increase in E7-specific IFNγ + T cells in three patients after vaccination. Reduction in total tumor size was observed in four patients, suggesting that Lm-LLO-E7 may have therapeutic effects in controlling cancer progression. The therapeutic potential demonstrated by the Lm-LLo-E7 vaccine has prompt the scientists to plan and design additional clinical trials to further determine the efficacy of this vaccine, including a phase II trial in patients with persistent, recurrent, loco-regional or metastatic anal cancer or HPV+ squamous cell carcinoma of the rectum (NCT02399813), a phase II trial in patients with HPV+ OPC before resection (NCT02002182), a phase I/II trial in patients with locally advanced or metastatic cervical or HPV+ head and neck cancer with or without MED14736 chemobile treatment (NCT02291055), and a phase II trial in patients with persistent or recurrent squamous or non squamous cell carcinoma of the cervix (NCT01266460).

A recent study tested the efficacy and safety of oral administration of GLBL101c, a bacterial vector-based therapeutic HPV vaccine. The Phase I/IIa study involved 17 patients with HPV16+ CIN3 lesions [32]. GLBL101c is generated from a recombinant *L. casei* that expresses a modified HPV16-E7 antigen, which is no longer carcinogenic [23]. The bacterial vector-based vaccine was administered to patients through ingestion after it was made into powder and enclosed into capsule form. None of the participating patients in this study experienced serious adverse effects. A significant increase in E7-specific cell-mediated immune responses in the cervical vaginal tract was observed in all patients who received the vaccine.

Other attenuated bacterial vectors can also be created through transformation with plasmids containing the genes of interest. For example, *Salmonella*, *Shigella*, and *Escherichia coli*, can deliver plasmid encoding genes of interest to APCs. Previous studies have tested the use of Salmonella for the delivery of HPV16 E7 protein or E7 epitopes to elicit E7-specific responses [33].

##### Viral vectors

Several viral vectors have been examined to deliver HPV E6 and E7 antigens including adenoviruses, adeno-associated viruses, alphaviruses, lentivirus, and vaccinia viruses [16, 34–45].

Vaccinia virus is an enveloped, double-stranded DNA virus belonging to the Poxvirus family. Vaccinia virus has a large genome, highly infectious nature, and low likelihood of unregulated integration of foreign DNA into its genome, making it a promising viral vector for vaccine delivery [46].

Several Modified Vaccinia Ankara (MVA)-based therapeutic HPV vaccines have been created and tested in recent years. TG4001 is a suspension of MVATG8042 particles consisting of attenuated recombinant MVA including sequences encoding modified HPV16 E6/E7 and human IL-2. The safety and efficacy of this vaccine was evaluated in 21 patients with HPV16-related CIN2/3 lesions [47]. Each patient received three subcutaneous injections, at 1-week intervals of TG4001 in their thigh. Most adverse effects were mild or moderate, including inflammation, pruritus, edema at the injection site, lymphadenopathy, fever, headache, asthenia, bone pain, and vaginal discharge. Furthermore, HPV16 DNA clearance was observed in 8 out of 10 responders, HPV16 mRNA clearance in 7, and no recurrence of high-grade lesions was observed for 12 months after treatment.

Another MVA-based vaccine, MVA E2 was created to deliver E2 proteins into vaccinated hosts rather than E6 and E7 [48]. The vaccine utilize the knowledge that E2 protein serve as inhibitor for the expression of E6 and E7 oncoproteins, and that the introduction of E2 into the host may suppress the activity of E6 and E7 in HPV-infected host, and subsequently reduce the transforming ability of the infected cells and the survivability of the malignant, HPV-associated tumor cells [9, 15]. In addition, E2 protein has been shown to arrest cell growth and induce apoptosis of cancer cells [49]. In addition, since some of the HPV-infected cells do not experience the lost of E2 gene during the transformation process (for review see [50–52]), MVA E2 vaccine may also lead to the generation of CD8+ T cells that can target the E2 antigen expressing, HPV-infected cells. Thus, MVA E2 may generate therapeutic antitumor effects against HPV-associated lesions through immunologic and biological mechanisms. MVA E2 was recently tested in a phase III clinical study for the treatment of HPV-induced ano-genital intraepithelial lesions [53], involving a total of 1356 patients (both male and female). Patients were injected locally at the lesion site or into visible lesions. The overall efficacy in this study for treating HPV-induced CIN lesions was around 90 % and all males showed complete eradication of lesions. Additionally, antibodies against HPV-E2 protein and MVA E2 vaccine were identified in the serum of all treated patients and cytotoxic T cell response that are specific to HPV-transformed cells were observed. The results of this clinical study demonstrate the therapeutic potential of the MVA E2 vaccine in treating HPV+ disease and potentially stimulating the immune system to target HPV-associated intraepithelial lesions.

TA-HPV is a live recombinant vaccinia virus vaccine that encodes oncoproteins E6 and E7 of both HPV type 16 and HPV18. TA-HPV was first used in clinical trial in eight patients with advanced stage cervical cancer [46]. In three patients’ TA-HPV induced an HPV-specific cytotoxic T cell response, and two patients were tumor free at 15 and 21 months after vaccination. In an additional clinical study using TA-HPV, 8 out of 29 patients displayed HPV specific serological responses, but HPV-specific cytotoxic T cell response was short lived [54]. Another clinical trial administered TA-HPV to 12 patients with HPV16+ vulval intraepithelial neoplasia (VIN) grade III and one patient with HPV16+ vaginal intraepithelial neoplasia (VAIN) grade II [55]. A reduction in HPV-associated lesion size was observed in patients as well as a significant increase in HPV16 E6/E7-specific T cell response.

As mentioned earlier, one challenge to using live vector-based therapeutic HPV vaccines is the generation of antibacterial or antiviral immune responses and neutralizing antibodies after initial vaccine exposure. Consequently, this limits the efficacy of multiple vaccine administrations. This issue was partially addressed by a former study, which showed that cyclooxygenase 2 (COX-2) inhibitors may prevent the production of neutralizing antibodies to vaccinia virus [56].

#### Peptide and protein-based vaccines

Peptide and protein that are derived from HPV antigens are processed by DCs and presented on either MHC class I or class II molecules to stimulate CD8+ or CD4+ T cell immune response [12, 57]. Furthermore, peptide and protein-based vaccines are safe, stable, and easy to produce.

##### Peptide-based vaccines

Although peptide-based vaccines are safe, stable, and easy to produce they have poor immunogenicity and require lipids or other adjuvants, such as chemokines, cytokines, and Toll-like receptor (TLR) ligands, to enhance vaccine potency [57]. These methods help improve the vaccine’s ability to activate innate and adaptive immunity and further boost CD8+ T cell responses (for review see [12]). Unfortunately, peptide-based vaccines are MHC specific, meaning that, for the vaccine to be effective the specific immunogenic epitopes of HPV antigens needs to be identified for each individual. Due to the MHC specificity required of peptide-based vaccines, they face certain challenges for large scale production and treatment of HPV-associated diseases [58]. One possible solution is the application of overlapping long-peptide vaccines. This method has been proven effective at inducing antigen-specific T cell responses in several preclinical models, (for review see [57, 58]).

HPV16 synthetic long-peptide vaccine (HPV16-SLP) and its therapeutic effects have been studied extensively in several clinical trials [59–61]. HPV16-SLP consists of both E6 and E7 overlapping peptides and Montanide ISA-51 as an adjuvant [62]. A recent placebo-controlled, double-blinded phase II study further investigated the capability of HPV16-SLP vaccines to establish long-term immunologic memory in patients with low-grade abnormalities of the cervix [63]. In this study 50 patients were randomly assigned to receive HPV16-SLP vaccination or placebo, followed by a vaccine or placebo booster one year later. Observed adverse effects included flu-like symptoms and injection site reactions. 97 % of vaccinated patients showed a significant HPV16-specific immune response and the study demonstrated that two low-dose vaccinations of HPV16-SLP could induce a robust HPV16-specific T cell response, lasting up to one year. Although clinical and virological responses were not the objectives of the study, clinical regression and viral clearance in several patients were observed.

Another study investigated whether HPV16-SLP vaccination combined with standard carboplatin and paclitaxel (CarboTaxol) chemotherapy could improve immunity in patients with cervical cancer [64]. In addition, this study sought to determine the time point in which immunity is optimized and vaccine administration is most effective. Participating patients all had advanced, recurrent, or metastatic cervical cancer but were not required to have HPV16+ tumors. Patients were divided into two cohorts. Six patients were recruited for the first cohort and were administered CarboTaxol treatment once every three weeks for a total of six CarboTaxol treatments to determine which time point immunity is optimized. Levels of myeloid cells dropped, reaching their lowest levels 1 to 2 weeks after the second chemotherapy cycle in patients receiving CarboTaxol treatment. Furthermore, the decrease in myeloid cells corresponded with an increase in lymphoid cells. Although the relative frequencies of CD4+ and CD8+ T cells did not change, T cell function was enhanced. 13 patients and 19 healthy donors participated in the second cohort, which looked at whether CarboTaxol mediates normalizing of circulating immune cell frequencies. 12 patients received a single vaccination of HPV16-SLP 2 weeks after their second (*n* = 11) or third (*n* = 1) chemotherapy cycle. During CarboTaxol treatment lymphocyte count did not change; however, the number of circulating leukocytes increased significantly. Myeloid and lymphoid cell counts in CarboTaxol patients reached levels close to normalized levels of healthy donors. In addition, HPV16-SLP vaccination enhanced T cell response in patients, which remained the same in 11 patients after six cycles of chemotherapy. Significant regressions of tumors in patients were not observed, and 1 patient died 11 weeks after vaccination due to disease progression. However, vaccination was well tolerated and most adverse events observed were localized, injection site reactions. Additional clinical studies are being conducted to continue to evaluate the therapeutic potential of HPV-16 SLP vaccine. These includes a phase I/II trial in HIV+ male patient with HPV-16+ AIN2/3 (NCT01923116), a phase II trial in patients with HPV-16+ incurable solid tumors in combination with Nivolumab (NCT02426892), and a phase I/II trial in female patients with HPV-16+ advanced or recurrent cervical cancer (NCT02128126).

PepCan is a therapeutic HPV vaccine consisting of four synthetic peptides covering HPV16 E6 and Candin, a novel adjuvant. The safety of PepCan was tested in a phase I clinical trial study in 31 patients with high grade squamous intraepithelial lesions (HSIL) [65]. PepCan was administered intradermally every three weeks at 50, 100, 250, 500ug per peptide dose in six patients. 12 weeks after the last injection a loop electric excision procedure was performed to remove lesion tissues. In the final dose portion of the phase I clinical trial an additional ten patients with biopsy-confirmed HSIL (any HPV type) were vaccinated with 50ug per peptide dose. Common adverse effects were mild to moderate injection site reactions, with no dose-limiting toxicities reported. The 50ug per peptide dosage demonstrated the best histological regression rate (50 % complete regression) and the most significant virological response (85 % viral clearance). Lastly, the viral load decreased in nine patients in whom HPV infection was detected at entry and exit. A new study is currently ongoing to further assess the efficacy of Pepcan + candin vaccination regimen in a phase II trial with cervical HSIL patients (NCT02481414).

Additionally, a phase I dose escalation trial using therapeutic HPV-peptide based vaccine with adjuvant Montanide and GMCSF (GL-0810) has been tested in five patients with recurrent/metastatic (RM) squamous cell carcinoma of the head and neck (SCCHN) [66]. GL-0810 was injected subcutaneously into participating patients. Overall, the vaccine was well tolerated with some adverse effects including erythema, pain, and itching at injection site. Four patients (80 %) with HPV16+ RM SCCHN generated T cell and antibody responses. Furthermore, no dose-limiting toxicities were observed. This trial demonstrated that GL-0810 is able to elicit an immune response and is well tolerated by patients with late stage SCCHN.

In addition to the vaccine candidates described above, a Phase I clinical trial has also been planned to evaluate the safety and therapeutic effect of PDS0101, a new therapeutic HPV vaccine candidate consist of Peptides from HPV-16 E6 and E7 as antigen and R-enantiomer of 1,2-dioleoyl-3-trimethylammonium-propane chloride as adjuvant, in female patients with high risk HPV infection or CIN1 (NCT02065973). Furthermore, a phase Ib/II trial has been planned to evaluate the therapeutic HPV vaccine DPX-E7, a HPV16-E7 aa11-19 nanomer peptide vaccine, in HLA-A*02 positive patients with incurable HPV16-related oropharyngeal, cervical, and anal cancer (NCT02865135).

##### Protein-based vaccines

One benefit to using protein-based vaccines is that they contain all human leukocyte antigen (HLA) epitopes. This avoids the limitation of MHC restriction, which is a setback to using peptide-based vaccines [67]. Protein-based vaccines, however, suffer from low immunogenicity and most are presented through the MHC class II pathway which activates the production of antibodies instead of generating a CTL response [58]. Strategies to overcome these problems focus on enhancing the MHC class I presentation. Adjuvants and immunostimulating molecules are added to protein-based vaccines to increase endogenous processing, to further increase protein uptake by MHC class I, and to effectively target to DCs, which increase MHC class I presentation and activation of CD8+ T cells [57].

Therapeutic HVP vaccine TA-CIN is a subunit vaccine encompassing HPV16 E6E7L2 fusion protein [68]. TA-CIN has been proven immunogenic and safe in several phase I/II clinical trials [69–71]. A phase II study tested the ability of TA-CIN to be administered with imiquimod, a topical immunomodulatory, to treat patients with high grade vulva intraepithelial neoplasia (VIN) [72]. A total of 19 patients received imiquimod 5 % cream and three intramuscular TA-CIN vaccinations (128ug/time) at 1-month intervals. Common adverse effects observed after imiquimod cream application included local inflammation, ulceration, malaise, and flu-like symptoms; however, no adverse effects associated with TA-CIN were observed. Twenty weeks after vaccination an increase in infiltrating CD8+ and CD4+ T cells were observed, and at 52 weeks complete regression of VIN was observed in 63 % of patients with 36 % of lesions showed HPV16 clearance. In this study, antigen-specific immune responses in patients correlated with lesion regression. Currently, another Phase I study has been planned to evaluate the safety and efficacy of TA-CIN in combination with adjuvant GPI-0100 in patients with HPV16 associated cervical cancer (NCT02405221).

GTL001 (ProCervix) is a therapeutic HPV protein-based vaccine that targets both HPV type 16 and 18 [73]. GTL001 is composed of recombinant HPV16 and HPV18 E7 proteins fused to catalytically inactive *Bordetella pertussis* CyaA expressed in *E. coli*. A phase I trial was conducted to examine the safety, tolerability, and immunogenicity of GTL001 in 47 women who had normal cytology but were positive for HPV16 or HPV18 infection. The participants were divided into four cohorts with two placebo sub-cohorts included within cohort three. Each patient was administered either 100ug or 600ug of GTL001 with imiquimod. Patients treated with GTL001 experienced injection site reactions including pain, swelling, induration, tenderness, and itching; however, the study demonstrated that vaccination with GTL001 was relatively safe. GTL001 induced a humoral response to CyaA in all subjects but anti-E7 antibodies were not induced. All patients showed similar immunogenicity and tolerability. Patients in cohort 4 (*n* = 9) who received 600ug GTL001 powder + imiquimod experienced the highest HPV16/18 clearance rate. However, in the recently press release by GENTICEL regarding their interim (18 months) results for the double-blinded, placebo controlled, phase II studies involving the use of GTL001 in 233 patients with HPV16/18+ normal or abnormal cytology (NILM, ASCUS, or LSIL) (NCT01957878), no significant difference in viral clearance was observed in HPV-16/18 positive patients receiving GTL001 vaccine versus placebo.

Additional clinical trials have been planned to test the potential of other therapeutic HPV protein vaccine candidates. One of such include the use of TVGV-1, a fusion protein of HPV-16 E7 antigen with ER targeting sequence, in a phase IIa trial with HPV induced cervical HSIL patients (NCT02576561).

#### Nucleic acid-based vaccines

##### DNA vaccines

DNA vaccines have risen in popularity as an attractive and potentially effective approach for antigen-specific immunotherapy. DNA vaccines are safe, stable, easy to produce and can sustain antigen expression in cells for longer durations than RNA vaccines or protein vaccines. Furthermore, they do not produce neutralizing antibodies, which permits repeated vaccination [67]. There is a potential risk that administration of DNA encoding HPV oncogenes E6 and E7 may lead to cellular transformation. This problem was addressed by modifying E6 and E7 DNA to result in subsequent expression of proteins that are incapable of oncogenic transformation [74]. DNA vaccines involve the injection of plasmid DNA that encodes the antigen of interest, in our case HPV E6 and E7, into the host’s cells.

DNA vaccines are often administered via intramuscular (IM) injection; however, myocytes are usually the cells that uptake the DNA after IM injection (for review see [67]). Although myocytes will express the target antigen, they are not professional APCs and therefore cannot activate a robust immune response [75]. DCs play an important role in presenting the antigen to naïve CD8+ cytotoxic T cells and do so through either phagocytosis and present exogenous antigen release from transfected myocytes on MHC class I through cross presentation, or direct transfection of DCs by vaccination leading to direct presentation to CD8+ T cells [76–78].

One limitation of DNA vaccines is that naked DNA is unable to amplify and spread from transfected cells to surrounding cells *in vivo* resulting in low immunogenicity. Consequently, several strategies have been developed to help overcome this hurdle (for review see [16]).

Therapeutic HPV DNA vaccines have undergone many clinical trials to evaluate the efficacy and safety of these vaccines. One such trial treated patients with HPV16-associated CIN2/3 lesions with heterologous prime-boost vaccination [79]. The DNA vaccine used in this study was pNGVL4a-sig/E7(detox)/HSP70, a plasmid encoding mutated form of HPV16-E7 linked to signal peptide and heat shock protein 70. pNGVL4a-sig/E7(detox)/HSP70 DNA vaccine was previously shown to enhance the HPV-16 E7 antigen-specific T cell mediated immune responses in a preclinical model [80]. This study also used TA-HPV vaccine as a booster. Twelve patients received two intramuscular injections of pNGVL4a-sig/E7(detox)/HSP70 boosted with TA-HPV, at 1 month intervals. Reported adverse effects included tenderness, local reaction at injection site, blister with drainage, erythema, and pruritus. This study suggested that local immune responses are ultimately responsible for the therapeutic effects against target lesions and can lead to better clinical outcomes. The prime boost regimen of pNGVL4a-sig/E7(detox)/HSP70 DNA + TA-HPV viral vector vaccines is continued to be examined in a phase I trial in combination with topical imiquimod application in HPV16+ CIN3 patients (NCT00788164).

A more recent clinical study was conducted to evaluate the safety, efficacy, and immunogenicity of pNGVL4a-CRT/E7(detox), a DNA plasmid vaccine [81]. pNGVL4a-CRT/E7(detox) was administered to 32 patients with HPV16-associated CIN2/3 either intradermal, intramuscularly, or directly into the cervical lesion (intralesionally) three times at four week intervals. This study demonstrated that pNGVL4a-CRT/E7(detox) was well tolerated in patients, and vaccination via intralesional injection elicited stronger immune response and induced more CD8+ T cells. Another phase I trial on pNGVL4a-CRT/E7(detox) DNA vaccine is still currently ongoing evaluating its safety and immunogenicity in patients with HPV16+ CIN2/3 lesions (NCT00988559).

A phase I clinical trial was conducted to evaluate the safety and efficacy of GX-188E, a therapeutic HPV DNA vaccine. The study was conducted in nine patients with high grade squamous intraepithelial lesions (HSIL/CIN3) [82]. GX-188E is a DNA vaccine engineered to express HPV16 and HPV18 proteins E6/E7 fused to the extracellular domain of Flt3L and signal sequence of plasminogen activator (tpa). Flt3L and tpa were included to enhance the potency of the vaccine through trafficking promotion and presentation of the fusion protein to the secretory pathway. Nine patients were administered GX-188E through intramuscular injection followed by electroporation to enhance immunogenicity. The results of this study demonstrated GX-188E to be safe and well tolerated by patients. Furthermore, all patients showed a statistically significant cellular immune response and three patients showed a weak antibody response against E7 protein. Genexine, Inc has planned two additional clinical trials on GX-188E, including a Phase II trial to be conducted in Eastern Europe targeting patients with HPV16/18+ CIN2, CIN2/3, or CIN3 lesions (NCT02596243), as well as a phase II trial to be conducted in South Korea targeting patients with HPV 16/18+ CIN3 lesions (NCT02139267).

An additional clinical study tested the therapeutic effects of HPV DNA vaccine VGX-3100. VGX-3100 is a combination of two plasmids encoding optimized HPV16 and 18 E6 and E7 antigens [83, 84]. VGX-3100 was administered through intramuscular injection followed by electroporation to 18 female patients who had been previously treated for CIN2/3 lesions [85]. Each patient received three rounds of vaccination, which was well tolerated with no observed dose-limiting toxicities. Adverse effects included injection site reaction, fever, pain during electroporation, and tenderness. Fourteen out of 18 patients (78 %) showed induced HPV-specific CD8+ T cells with full cytolytic function, 17 out of 18 (94 %) of patients had increased HPV16 E7 antibody titers, and all patients had increased HPV18 E7 antibody titers. Additionally, 12 patients (67 %) had increased HPV16 E6 antibody titers, and seven (39 %) patients had increased HPV18 E6 titers. These results exhibited the potential of VGX-3100 to induce a robust antigen-specific immune response and contribute to the eradication of HPV-infected cells and lesion regression. Furthermore, the results of this phase I study encouraged a follow up phase IIb clinical trial to further investigate the therapeutic efficacy of VGX-3100 DNA vaccine on CIN 2/3 lesions in a randomized, double-blind, placebo-controlled study [86]. This study correspondingly demonstrated that patients vaccinated with VGX-3100 showed greater HPV-specific T cell and humoral responses. Inovio Pharmaceuticals has recently designed a new vaccine formulation INO-3112, incorporating the proprietary immune activator expressing IL-12 (INO-9012) with the VGX-3100 DNA vaccine. This formulation is being tested in a phase I/IIA trial in patients with HPV associated head and neck squamous cell carcinoma (NCT02163057), a phase I/IIA trial in female patients with new, recurrent, or persistent cervical cancer (NCT02172911), as well as a phase II trial in patients with locally advanced cervical cancer who received standard of care chemoradiation (NCT02501278).

##### RNA vaccines

Naked RNA replicon vaccines can be derived from several RNA viruses including Sindbis virus, Venezuela Equine Encephalitis virus, and SFV [34, 87, 88]. RNA replicons are capable of self-replication, which can lead to a sustained level of antigen expression and increased immunogenicity. Furthermore, RNA replicon vectors do not form viral particles, meaning they will not lead to the generation of neutralizing antibodies, permitting repeated administration. RNA replicons are also highly advantageous vaccination methods because they do not run the risk of chromosomal integration and cellular transformation that can occur by using DNA vaccines. One drawback to RNA replicons, however, is their low stability. One attempt to overcome this issue combined RNA replicons and DNA vaccine into DNA-launched RNA replicons, which are also referred to as ‘suicidal DNA.’ These ‘suicidal DNA’ trigger apoptosis in cells that uptake the injected DNA to prevent further integration and transformation of transfected cells [89]. However, because it will elicit apoptosis in transfected cells, including DCs, this approach has led to poor immunogencity. Several strategies have been created to address this issue. One example is the inclusion of genes encoding anti-apoptotic protein into ‘suicidal DNA’ to enhance survival of transfected APCs [90]. Another strategy employed to overcome apoptosis is the use of flavivirus Kunjin (KUN) vector to deliver replicons. Since KUN does not induce apoptosis in transfected cells, it enables the direct presentation by transfected DCs [91, 92]. Although RNA replicon vaccines have shown promising results in preclinical models and in other types of cancer setting [93], RNA vaccines targeting HPV antigens and HPV-associated diseases have not yet been explored in the clinical setting.

#### Whole cell-based vaccines

##### Dendritic cell-based vaccines

DCs play an important role in immune system regulation, and they are commonly identified as the most efficient professional APCs [67]. DC-based vaccines have grown as the biological knowledge of DC and methods for preparing DCs *ex vivo* have improved. DC-based HPV vaccines involve loading the DCs with HPV antigens *ex vivo* and delivering those DCs to the infected host [94–98]. One benefit of DC-based vaccines is that DCs can serve as natural adjuvants to increase the potency of antigen-specific immunotherapy against cancer (for review see [99]). Because T cell-mediated apoptosis may limit DC lifespans, some strategies have been developed to prolong the survival of DCs. One such strategy is to transfect DCs with siRNAs targeting pro-apoptotic molecules. These strategies have been shown to generate a greater antigen-specific CD8+ T cell activation and antitumor effects in mice [95, 97, 100].

Due to the therapeutic potential demonstrated by DC-based vaccines in preclinical models, further clinical trials were developed to test DC-based vaccine efficacy in humans. One such study was conducted to evaluate safety, toxicity, and immunogenicity of a DC-based vaccine in ten patients with stage Ib or IIa cervical cancer [101]. In this phase I, dose escalation trial autologous DCs were obtained from patients and pulsed with full length HPV16/18-E7 oncoprotein and keyhole limpet hemocyanin (KLH). The patients were then vaccinated with the pulsed DCs through subcutaneous injection. The DC-based vaccine used in the study was reported safe and well tolerated by patients, and resulted in minor local reactions, including erythema, swelling, and pruritus. There was an increase in HPV-specific humoral response and an increase in E7-specific CD4+ T cells in patients after vaccination.

An additional phase I clinical trial investigated the toxicity and immunogenicity of a DC-based vaccine in 14 patients with HPV+ advanced, recurrent cervical cancer [102]. Patients were separated into three treatment groups: saline only control, unprimed mature DC, and autologous tumor lysate primed mature DC. DCs were collected from each patient and pulsed with or without tumor lysate obtained from the same patient. Grade 0 and grade 1 toxicity, including itching at vaccination site, fever, chills, abdominal discomfort, and vomiting, were observed in three of the 14 patients, implying that the DC-based vaccine was well tolerated. No statistically significant increase in lymphocyte proliferation was observed in all patient groups.

In congruence with other vaccine types, DC-based vaccines have several limitations. DC-based vaccines are technically taxing, thus making them a poor choice for large-scale production. In addition, varying culturing techniques may lead to inconsistent vaccine quality and lack of standard criteria for vaccine evaluation. Lastly, the most effective administration route for DC-based vaccines has not yet been determined.

##### Tumor cell-based vaccines

To create tumor cell-based vaccines, tumor cells are isolated and manipulated *ex vivo* to express immune modulatory proteins, which can further enhance their immunogenicity *in vivo*. Cytokine genes IL-2, IL-12, and granulocyte macrophage colony stimulating factor (GMCSF) have been used to induce differentiation of naïve T cells into effector or helper T cells and to stimulate granulocyte production in HPV tumor cell-based vaccines in mice with HPV16 induced tumors [103, 104]. One advantage to tumor cell vaccines is that tumor antigens do not need to be well defined; therefore, these vaccines may be able to cover a wider range of tumor antigens. Since HPV has well-known tumor-specific antigens, tumor cell-based vaccines may not be the most practical immunotherapy for HPV-associated cancers. In addition, tumor cell-based vaccines run the risk of implanting new cancers in patients. Due to the nature of these vaccines and their potential risks, the potency and purity of each vaccine must be individually tailored, making production expensive and time consuming. For these reasons tumor cell-based vaccines targeted against HPV have not yet been developed and tested in clinical studies.

## Conclusion

The identification of high-risk HPV as the etiological factor for many diseases provides justification for the development of therapeutic HPV vaccines. The recent developments in the field as well as those discussed in this review have helped contribute to the foundational movement to eradicate HPV and HPV-associated diseases and malignancies. In this review we discussed the various methods of targeting HPV oncoproteins E6 and E7, which represent tumor-specific antigens and excellent targets for therapeutic HPV vaccines. Based on our own previous studies, and those conducted by other investigators in the field, we believe that the current therapeutic HPV vaccines mentioned in this review each possess advantages and limitations. Additional clinical studies are still necessary to further verify the antitumor efficacy of therapeutic HPV vaccines.

With continued efforts to improve and develop therapeutic treatment strategies, we anticipate the continued success of therapeutic HPV vaccines over the next few years, and beyond. We believe that therapeutic HPV vaccines will become clinically available in the near future and be offered alongside other available therapies for the control of HPV-associated diseases.

## Abbreviations

APC: Antigen presenting cell
B7-H1: B7 homolog-1
CarboTaxol: Carboplatin and paclitaxel
CIN: Cervical intraepithelial neoplasia
COX-2: Cyclooxygenase 2
CRT: Calreticulin
CTL: Cytotoxic T lymphocyte
DC: Dendritic cell
ER: Endoplasmic reticulum
GMCSF: Granulocyte macrophage colony stimulating factor
HBsAg: Hepatitis B virus surface antigen
HDACi: Histone deacetylase inhibitor
HLA: Human leukocyte antigen
HPV: Human papillomavirus
HPV16-SLP: HPV16 synthetic long-peptide vaccine
IDLV: Integrase defective lentiviral vector
IDO: Indoleamine 2,3-dioxygenase enzyme
IFN: Interferon
IFNγ: IFN-gamma
IM: Intramuscular
ISG15: Interferon-stimulating gene 15
LLO: Listeriolysin O
MHC: Major histocompatibility complex
MICA/B: MHC class I polypeptide-related sequence A and B
PBMC: Peripheral blood mononuclear cells
RM: Recurrent/metastatic
rSFV: Recombinant Semliki Forest virus
SCCHN: Squamous cell carcinoma of the head and neck
SFV: Semliki Forest virus
STAT3: Signal transducer and activator of transcription 3
TCR: T cell receptor
TGFβ: Tumor growth factor-beta
TLR: Toll-like receptor
tpa: Signal sequence of plasminogen activator
VAIN: Vaginal intraepithelial neoplasia
VIN: Vulval intraepithelial neoplasia
